# Supplementary material for: Hsp90 buffers behavioral variability by regulating Pdf transcription in clock neurons of Drosophila melanogaster
Source: PLoS Genet. 2026 Feb 17;22(2):e1012044. doi: 10.1371/journal.pgen.1012044 (PMC12952617; doi:10.1371/journal.pgen.1012044)
Supplement: S1 Fig — A) Population Activity (top) and HMM-implied percentage of time spent in the active state (bottom) of Hsp83 heterozygous (Hsp83 mutant/+) flies in LD. B) Population Activity (top) and HMM-implied percentage of time spent in the active state (bottom) of Hsp83 heterozygous (Hsp83 mutant/+) flies in DD (repeats: 4). See also Tables 1, 2, S2 and S3. (DOCX) [file pgen.1012044.s001.docx]

**
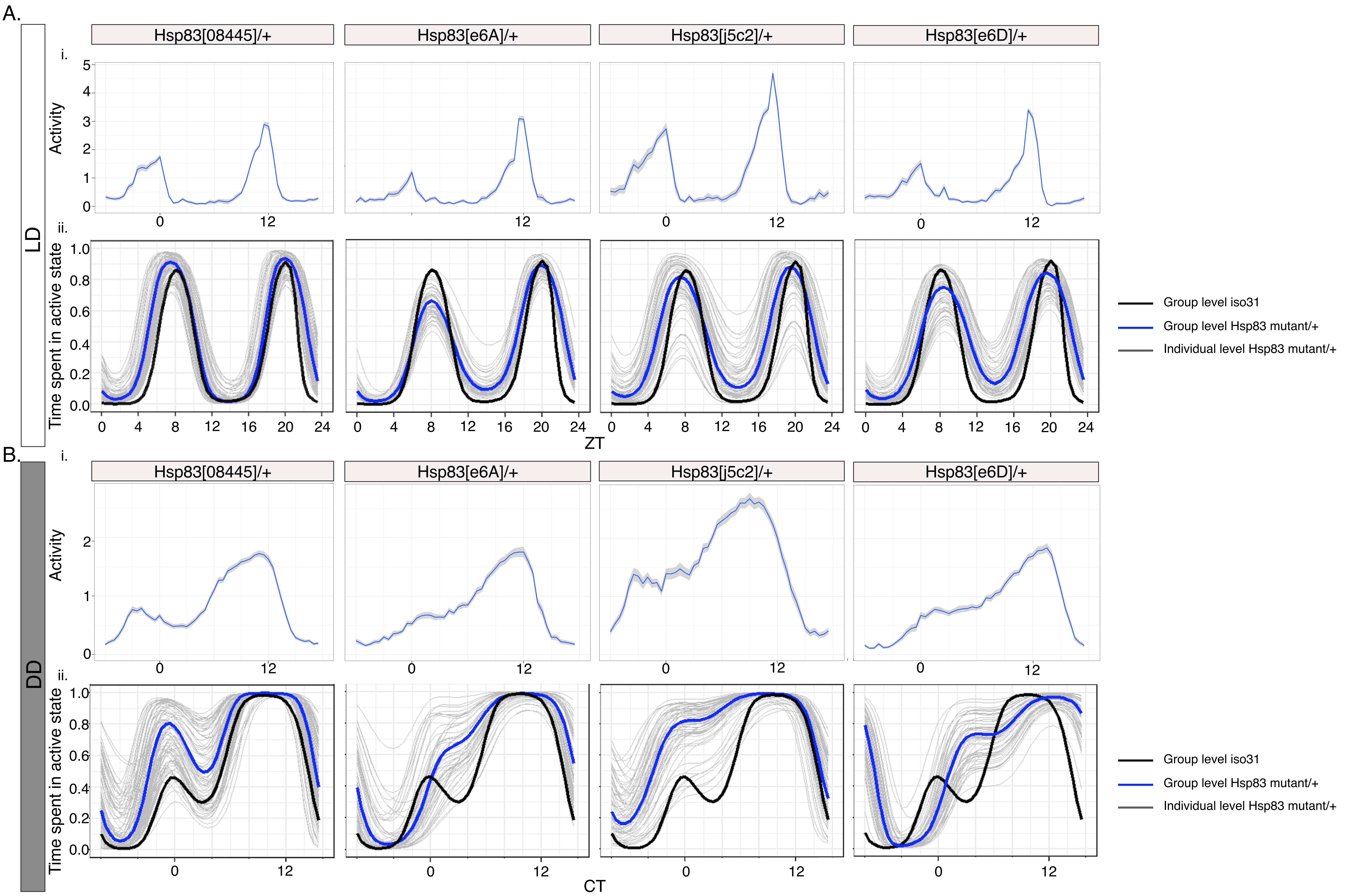
**

**S1 Fig . Heterozygous Hsp83 loss of function does not affect behavior in LD and DD.**

A) Population Activity (top) and HMM-implied percentage of time spent in the active state (bottom) of *Hsp83* heterozygous (*Hsp83* mutant/+) flies in LD (repeats: 4). B) Population Activity (top) and HMM-implied percentage of time spent in the active state (bottom) of *Hsp83* heterozygous (*Hsp83* mutant/+) flies in DD (repeats: 4). See also Tab 1, 2, S2, S3.
