# Supplementary material for: Hsp90 buffers behavioral variability by regulating Pdf transcription in clock neurons of Drosophila melanogaster
Source: PLoS Genet. 2026 Feb 17;22(2):e1012044. doi: 10.1371/journal.pgen.1012044 (PMC12952617; doi:10.1371/journal.pgen.1012044)
Supplement: S2 Fig — A) Circular phase plot comparing Hsp8308445 mutant flies with or without expression of UAS-Hsp83 in all clock neurons (repeats: 4–5). B) Circular phase plots of trans-heterozygous Pdf01/Hsp8308445 (left) and Pdf01/Hsp83e6A (right) mutant flied and their respective controls./+) (repeats: 2). Arrhythmic flies were excluded from the peak analysis. Colored lines represent the median phase of each group, while the length of the vector represents the circular standard deviation of the corresponding group (1/circular SD). Watson’s goodness of fit test and pairwise equal kappa (κ) test **** p < .0001, *** p < .001, ** p < .01, * p < .05, ns p > .05. (DOCX) [file pgen.1012044.s002.docx]

**
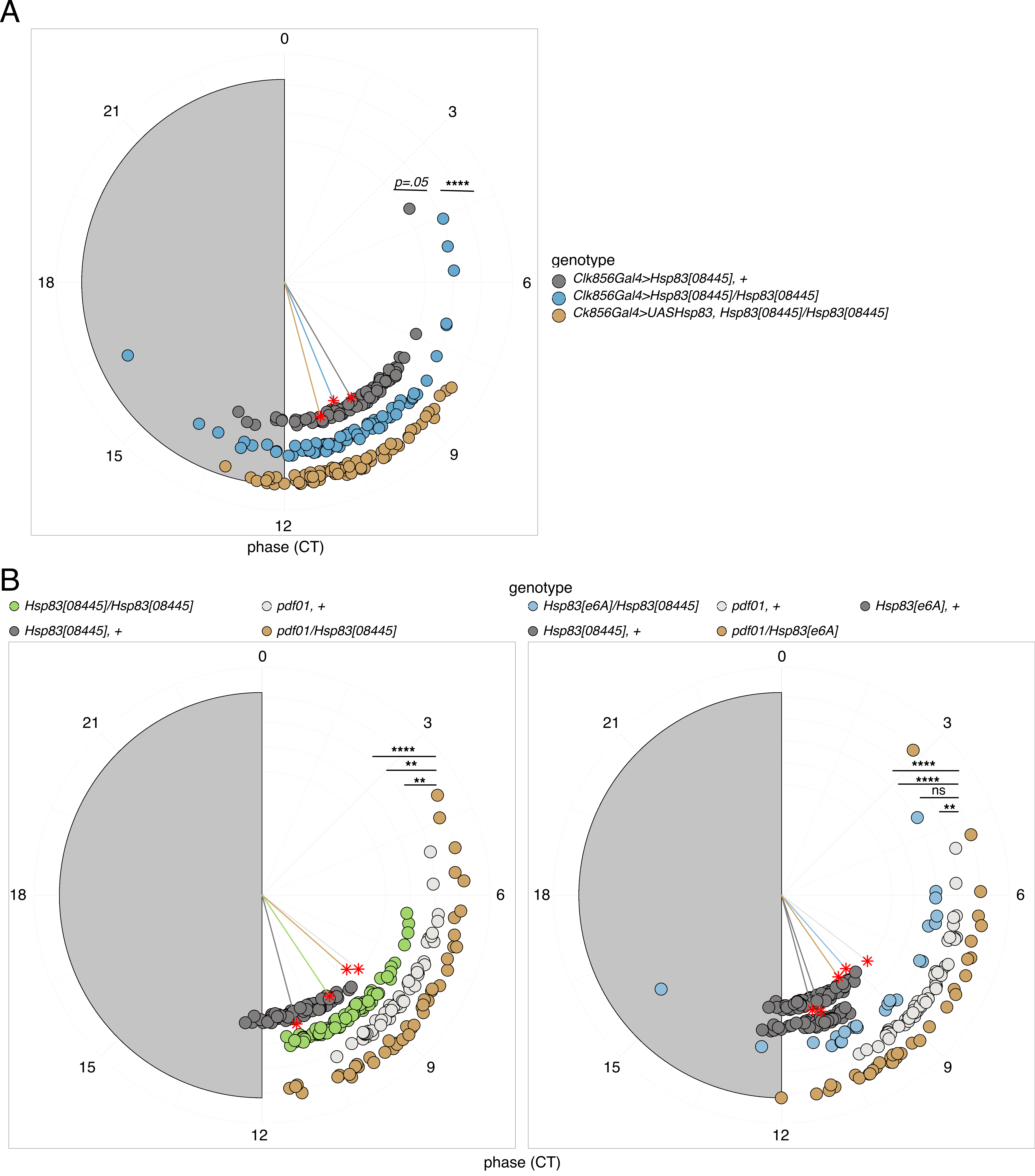
**

**S2 Fig. Clock neuron-specific rescue of *Hsp83* mutants and genetic interaction with *Pdf***

A) Circular phase plot comparing *Hsp83^08445^* mutant flies with or without expression of *UAS-Hsp83* in all clock neurons (repeats: 4-5). B) Circular phase plots of trans-heterozygous *Pdf^01^*/*Hsp83^08445^* (left) and *Pdf^01^*/*Hsp83^e6A^* (right) mutant flied and their respective controls. /+) (repeats: 2). Arrhythmic flies were excluded from the peak analysis and are not shown in the figure. Colored lines represent the median phase of each group, while the length of the vector represents the circular standard deviation of the corresponding group (1/circular SD). Watson's goodness of fit test and pairwise equal kappa (κ) test **** *p* < .0001, *** *p* < .001, ** *p* < .01, * *p* < .05, ns *p* >.05.
