# Supplementary material for: Hsp90 buffers behavioral variability by regulating Pdf transcription in clock neurons of Drosophila melanogaster
Source: PLoS Genet. 2026 Feb 17;22(2):e1012044. doi: 10.1371/journal.pgen.1012044 (PMC12952617; doi:10.1371/journal.pgen.1012044)
Supplement: S3 Fig — A) Quantification of the RFP levels in Pdf-Red; + / + compared to Pdf-red; Hsp8308445/Hsp8308445 flies for the s-LNv, dorsal projection and l-LNv, using Estimation Statistics. B) Quantification of RFP levels in the cell bodies (left) and projections (right) of CCAP neurons (CCAP-Gal4 > UAS-myr-mRFP1) either in a wild type background or with the Hsp8308445 mutation C) RFP expression in the CCAP+ neurons using the CCAP-Gal4. Scale bar: 20μm. D) Representative images of anti-PDF staining of the s-LNv, dorsal projections, and l-LNv in Hsp83 knock out (Clk856-Gal4 > UAS-Cas9, hsp83 sgRNA) and control flies (hsp83 sgRNA/+). Scale bar: 20μm. E) Left: Representative images of anti-VRI staining in the l-LNv and s-LNv of iso31 and Hsp8308445/Hsp8308445 flies. Scale bar: 20μm. Right: Quantification of the intensity of VRI staining in the s-LNv and l-LNv in 8 hemispheres, using Estimation Statistics. (DOCX) [file pgen.1012044.s003.docx]

**
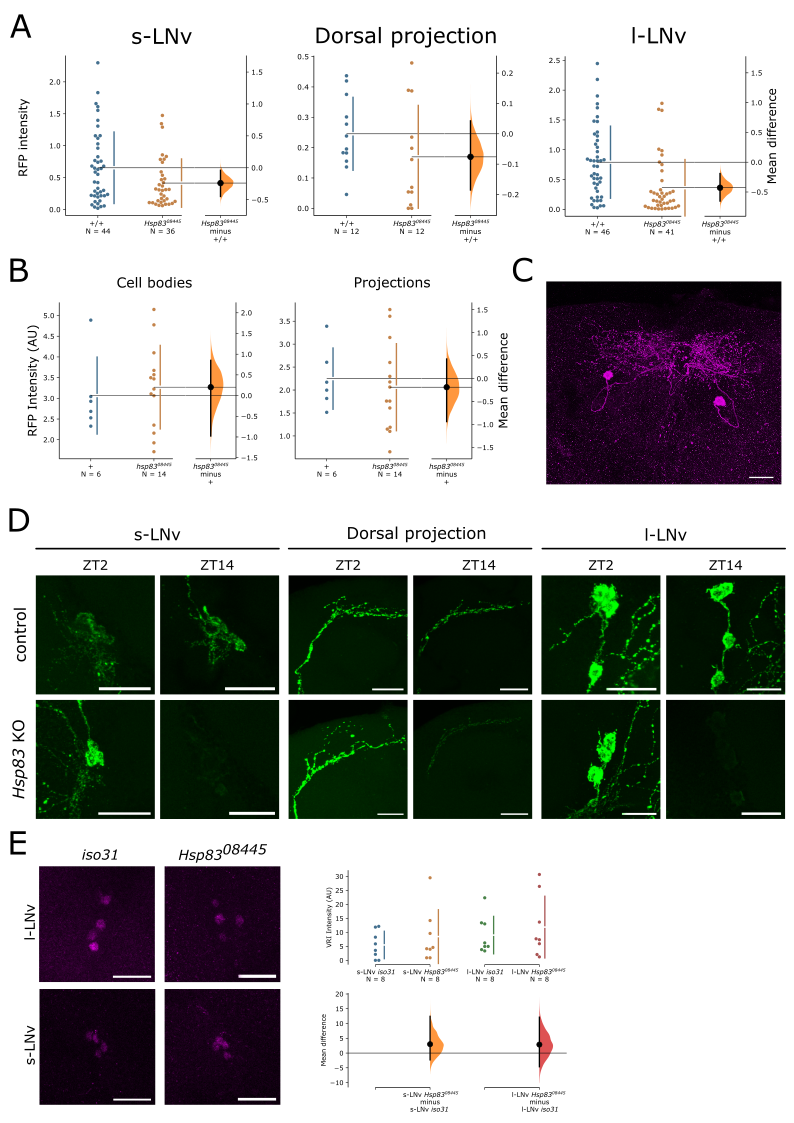
**

**S3 Fig. Hsp83 depletion affects PDF mRNA and protein levels.**

A) Quantification of the RFP levels in *Pdf-Red; +/+* compared to *Pdf-red; Hsp83^08445^/Hsp83^08445^* flies for the s-LNv, dorsal projection and l-LNv, using Estimation Statistics. B) Quantification of RFP levels in the cell bodies (left) and projections (right) of CCAP neurons (*CCAP-Gal4 > UAS myr mRFP1*) either in a wild type or *Hsp83^08445^* mutant background. C) RFP expression in the CCAP+ neurons driven by *CCAP-Gal4*. Scale bar: 20μm. D) Representative images of anti-PDF staining of the s-LNv, dorsal projections, and l-LNv in *Hsp83* knock out (*Clk856-Gal4 > UAS-Cas9, hsp83 sgRNA*) and control flies (*hsp83 sgRNA/+*). Scale bar: 20μm. E) Left: Representative images of anti-VRI staining in the l-LNv and s-LNv of *iso31* and *Hsp83^08445^/Hsp83^08445^* flies. Scale bar: 20μm. Right: Quantification of the intensity of VRI staining in the s-LNv and l-LNv in 8 hemispheres, using Estimation Statistics.
