# Supplementary material for: Hsp90 buffers behavioral variability by regulating Pdf transcription in clock neurons of Drosophila melanogaster
Source: PLoS Genet. 2026 Feb 17;22(2):e1012044. doi: 10.1371/journal.pgen.1012044 (PMC12952617; doi:10.1371/journal.pgen.1012044)
Supplement: S1 Table — (DOCX) [file pgen.1012044.s004.docx]

**S1 Table.** **Viability of *Hsp83* allelic combinations.**

|  | | Males | | | | |
| --- | --- | --- | --- | --- | --- | --- |
|  |  | *Df(3L)BSC672* | *Hsp83^08445^* | *Hsp83^e6A^* | *Hsp83^e6D^* | *Hsp83^j5c2^* |
|  | *iso31* | + | + | + | + | + |
|  | *Hsp83^08445^* | + | + | + | + | + |
| Females | *Hsp83^e6A^* | - | + | - | - | - |
|  | *Hsp83^e6D^* | - | + | - | - | - |
|  | *Hsp83^j5c2^* | - | + | - | - | - |
|  | *Df(3L)BSC672* | - | + | - | - | - |
|  | | Females | | | | |
|  |  | *Df(3L)BSC672* | *Hsp83^08445^* | *Hsp83^e6A^* | *Hsp83^e6D^* | *Hsp83^j5c2^* |
|  | *iso31* | + | + | + | + | + |
|  | *Hsp83^08445^* | + | + | + | + | + |
| Males | *Hsp83^e6A^* | - | + | - | - | - |
|  | *Hsp83^e6D^* | - | + | - | - | - |
|  | *Hsp83^j5c2^* | - | + | - | - | - |
|  | *Df(3L)BSC672* | - | + | - | - | - |
|  | | Males | | | | |
|  |  | *iso31* | *Hsp83 sgRNA* | *TimGal4:27; UASCas9* | *Clk856Gal4; UASCas9* | *PdfGal4; UASCas9* |
| Females | *iso31* | + | + | + | + | + |
|  | *Hsp83 sgRNA* | + | + | - | + | + |
|  | | Females | | | | |
|  |  | *iso31* | *Hsp83 sgRNA* | *TimGal4:27; UASCas9* | *Clk856Gal4; UASCas9* | *PdfGal4; UASCas9* |
| Males | *iso31* | + | + | + | + | + |
|  | *Hsp83 sgRNA* | + | + | - | + | + |

*Note:* Viability is indicated with a + and crosses producing no trans-heterozygous mutant offspring are indicated with -.
