# Supplementary material for: Hsp90 buffers behavioral variability by regulating Pdf transcription in clock neurons of Drosophila melanogaster
Source: PLoS Genet. 2026 Feb 17;22(2):e1012044. doi: 10.1371/journal.pgen.1012044 (PMC12952617; doi:10.1371/journal.pgen.1012044)
Supplement: S2 Table — (DOCX) [file pgen.1012044.s005.docx]

**S2 Table. Rhythmic power of locomotor activity in LD.**

| Genotype | N. | RS ± SEM |
| --- | --- | --- |
| *iso31* | 70 | 2.0±0.06 |
| *Hsp83^08445^/+* | 86 | 2.0 ±0.04 |
| *Hsp83^08445^/Hsp83^08445^* | 75 | 1.7±0.05 |
| *Hsp83^e6A^/+* | 51 | 1.8±0.05 |
| *Hsp83^e6A^/Hsp83^08445^* | 34 | 1.6±0.11 |
| *Hsp83^e6D^/+* | 60 | 2.0 ± 0.07 |
| *Hsp83^e6D^/Hsp83^08445^* | 65 | 1.7 ± 0.06 |
| *Hsp83^j5c2^/+* | 51 | 2.0 ± 0.05 |
| *Hsp83^j5c2^/Hsp83^08445^* | 57 | 1.6 ± 0.09 |
| *Hsp83 sgRNA/+* | 48 | 2.5 ± 0.09 |
| *Clk856-Gal4>UAS-Cas9, +* | 73 | 2.0 ± 0.07 |
| *Clk856-Gal4>UAS-Cas9, Hsp83 sgRNA* | 67 | 1.9 ± 0.08 |
| *Pdf-Gal4>UAS-Cas9,+* | 69 | 2.2 ± 0.07 |
| *Pdf-Gal4>UAS-Cas9, Hsp83 sgRNA* | 75 | 1.8 ± 0.08 |
