# Supplementary material for: Hsp90 buffers behavioral variability by regulating Pdf transcription in clock neurons of Drosophila melanogaster
Source: PLoS Genet. 2026 Feb 17;22(2):e1012044. doi: 10.1371/journal.pgen.1012044 (PMC12952617; doi:10.1371/journal.pgen.1012044)
Supplement: S3 Table — (DOCX) [file pgen.1012044.s006.docx]

**S3 Table. Circular Phase plot and HMM analysis reveal increased variability of evening activity phase after HSP83 depletion in constant darkness (DD)**

| Genotype | N | Circular statistics | | | HMM |
| --- | --- | --- | --- | --- | --- |
|  |  | Median phase | *µ* ± SD | *κ* | SD of time spent in the active state |
| *iso31* | 69 | 10.2 | 10.3 ± 0.73 | 27.95 | 0.02 |
| *Hsp83^08445^/+* | 85 | 11.0 | 10.9 ± 0.72 | 28.48 | 0.01 |
| *Hsp83^08445^/Hsp83^08445^* | 73 | 9.7 | 9.7 ± 1.10 | 12.55 | 0.19 |
| *Hsp83^e6A^/+* | 50 | 10.7 | 10.9 ± 0.64 | 36.35 | 0.03 |
| *Hsp83^e6A^/Hsp83^08445^* | 25 | 9.2 | 9.1 ± 2.32 | 3.31 | 0.25 |
| *Hsp83^e6D^/+* | 59 | 12.0 | 11.9 ± 1.00 | 15.19 | 0.08 |
| *Hsp83^e6D^/Hsp83^08445^* | 63 | 12.0 | 11.8 ± 1.34 | 8.64 | 0.19 |
| *Hsp83^j5c2^/+* | 50 | 9.2 | 9.2 ± 1.02 | 14.86 | 0.03 |
| *Hsp83^j5c2^/Hsp83^08445^* | 54 | 9.2 | 8.9 ± 1.43 | 7.65 | 0.06 |
| *Hsp83 sgRNA/+;* | 129 | 10.5 | 10.2 ± 1.09 | 12.83 | - |
| *UAS-Cas9, Hsp83sgRNA* | 84 | 9.2 | 9.1 ± 1.85 | 4.80 | - |
| *Clk856-Gal4>UASCas9,+* | 71 | 9.7 | 9.8 ± 0.97 | 15.91 | - |
| *Clk856-Gal4>UAS-Cas9, Hsp83sgRNA* | 39 | 9.2 | 9.5 ± 3.14 | 2.09 | - |
| *Pdf-Gal4>UAS-Cas9,+* | 142 | 9.5 | 9.6 ± 0.85 | 20.51 | - |
| *Pdf-Gal4>UAS-Cas9, Hsp83 sgRNA* | 118 | 11.0 | 10.8 ± 2.27 | 3.42 | - |
| *Clk856 Gal4 > Hsp83^08445^/+* | 82 | 10.0 | 10.1 ± 1.34 | 8.64 | - |
| *Clk856 Gal4 >*  *Hsp83^08445^/ Hsp83^08445^* | 75 | 10.5 | 10.4 ± 1.78 | 5.17 | - |
| *Clk856 Gal4 >UAS-Hsp83, Hsp83^08445^/ Hsp83^08445^* | 85 | 11.0 | 10.8 ± 1.06 | 13.42 | - |
| *Pdf01/+* | 54 | 8.5 | 8.3 ± 1.10 | 12.61 | - |
| *Pdf01/ Hsp83^08445^* | 44 | 8.7 | 8.5 ± 1.82 | 4.94 | - |
| *Pdf01/ Hsp83^e6A^* | 34 | 9.7 | 8.99 ± 1.96 | 4.36 | - |

Note: *µ*: circular phase mean in circadian time; *κ*: concentration parameter of the circular distribution; SD: standard deviation.
