# Supplementary material for: Hsp90 buffers behavioral variability by regulating Pdf transcription in clock neurons of Drosophila melanogaster
Source: PLoS Genet. 2026 Feb 17;22(2):e1012044. doi: 10.1371/journal.pgen.1012044 (PMC12952617; doi:10.1371/journal.pgen.1012044)
Supplement: S4 Table — (DOCX) [file pgen.1012044.s007.docx]

**S4 Table. Number of PDF projections, s-LNvs and l-LNvs neurons per hemisphere in cell-specific *Hsp83* knock-out and parental control.**

| Neurons | Genotype | ZT | # hemisphere | # neurons per hemisphere (mean±SD) | % Hemispheres with abnormalities  (n. abnormal projections/total hemipheres) |
| --- | --- | --- | --- | --- | --- |
| s-LNv | *Hsp83 sgRNA/+* | 02 | 17 | 2.5 ± 0.72 | - |
|  |  | 14 | 21 | 3.4 ± 0.74 | - |
|  | *Clk856-Gal4>UAS-Cas9, Hsp83 sgRNA* | 02 | 28 | 2.7 ± 1.12 | - |
|  |  | 14 | 14 | 2.6 ± 1.01 | - |
| l-LNv | *Hsp83 sgRNA/+* | 02 | 22 | 3.6 ± 0.50 | - |
|  |  | 14 | 24 | 3.6 ± 0.49 | - |
|  | *Clk856-Gal4>UAS-Cas9, Hsp83 sgRNA* | 02 | 32 | 3.0 ± 0.97 | - |
|  |  | 14 | 17 | 2.8 ± 0.88 | - |
| PDF dorsal projections | *Hsp83 sgRNA/+* | - | 56 | - | 0.04% (2/56) |
|  | *Clk856-Gal4>UAS-Cas9, Hsp83 sgRNA* | - | 53 | - | 0.04% (2/53) |
