# Supplementary material for: Hsp90 buffers behavioral variability by regulating Pdf transcription in clock neurons of Drosophila melanogaster
Source: PLoS Genet. 2026 Feb 17;22(2):e1012044. doi: 10.1371/journal.pgen.1012044 (PMC12952617; doi:10.1371/journal.pgen.1012044)
Supplement: S5 Table — (DOCX) [file pgen.1012044.s008.docx]

**S5 Table.** **Watson's goodness of fit test to determine Von Mises distribution.**

|  | Watson's goodness of fit test | | | Fig. |
| --- | --- | --- | --- | --- |
| Parameters | U^2^ | p-value | Signif. | Fig. |
| iso31 | 4.69 | 0.52 | ns | 2 |
| *Hsp83^08445^/+* | 5.78 | 0.54 | ns |  |
| *Hsp83^08445^/ Hsp83^08445^* | 4.43 | 0.48 | ns |  |
| *Hsp83^e6A^/+* | 3.53 | 0.45 | ns |  |
| *Hsp83^e6A^/ Hsp83^08445^* | 1.02 | 0.57 | ns |  |
| *Hsp83^e6D^/+* | 3.75 | 0.33 | ns |  |
| *Hsp83^e6D^/ Hsp83^08445^* | 3.66 | 0.21 | ns |  |
| *Hsp83^j5c2^/+* | 3.12 | 0.57 | ns |  |
| *Hsp83^j5c2^/ Hsp83^08445^* | 3.12 | 0.13 | ns |  |
| *Hsp83 sgRNA/+* | 7.97 | 0.17 | ns | 4 |
| *UAS-Cas9, Hsp83sgRNA* | 3.91 | 0.52 | ns |  |
| *Clk856-Gal4> UAS-Cas9, +* | 4.47 | 0.55 | ns |  |
| *Clk856-Gal4 > UAS-Cas9, Hsp83sgRNA* | 1.09 | 0.60 | ns |  |
| *Pdf-Gal4 > UAS-Cas9, +* | 9.25 | 0.46 | ns |  |
| *Pdf-Gal4 > UAS-Cas9, Hsp83sgRNA* | 4.85 | 0.31 | ns |  |
| *Clk856 Gal4 > Hsp83^08445^/+* | 4.64 | 0.35 | ns | S2A |
| *Clk856 Gal4 > Hsp83^08445^/ Hsp83^08445^* | 3.75 | 0.26 | ns |  |
| *Clk856 Gal4 >UAS-Hsp83, Hsp83^08445^/ Hsp83^08445^* | 5.20 | 0.49 | ns |  |
| *Pdf01/+* | 3.29 | 0.49 | ns | S2B |
| *Pdf^01^/ Hsp83^08445^* | 2.09 | 0.58 | ns |  |
| *Pdf^01^/ Hsp83^e6A^* | 1.59 | 0.49 | ns |  |

**** *p* < .0001, *** *p* < .001, ** *p* < .01, * *p* < .05, ns *p* >.05.
