# Supplementary material for: Hsp90 buffers behavioral variability by regulating Pdf transcription in clock neurons of Drosophila melanogaster
Source: PLoS Genet. 2026 Feb 17;22(2):e1012044. doi: 10.1371/journal.pgen.1012044 (PMC12952617; doi:10.1371/journal.pgen.1012044)
Supplement: S6 Table — (DOCX) [file pgen.1012044.s009.docx]

**S6 Table.** **Pairwise equal kappa (*κ*) test with adjusted p-value using Bonferroni method.**

| Group 1 | Group 2 | χ^2^ | p-value | Signif. | Fig. |
| --- | --- | --- | --- | --- | --- |
| iso31 | *Hsp83^08445^/+* | 0.01 | 1.0 | ns | 2 |
|  | *Hsp83^08445^/ Hsp83^08445^* | 11.06 | 0.03 | * |  |
|  | *Hsp83^e6A^/+* | 0.93 | 1.0 | ns |  |
|  | *Hsp83^e6A^/ Hsp83^08445^* | 52.96 | < 0.0001 | **** |  |
|  | *Hsp83^e6D^/+* | 5.98 | 0.52 | ns |  |
|  | *Hsp83^e6D^/ Hsp83^08445^* | 22.24 | <0.001 | *** |  |
|  | *Hsp83^j5c2^/+* | 5.99 | 0.52 | ns |  |
|  | *Hsp83^j5c2^/ Hsp83^08445^* | 25.62 | <0.001 | *** |  |
| *Hsp83^08445^/+* | *Hsp83^08445^/ Hsp83^08445^* | 13.28 | <0.01 | ** |  |
|  | *Hsp83^e6A^/ Hsp83^08445^* | 61.16 | < 0.0001 | **** |  |
|  | *Hsp83^e6D^/ Hsp83^08445^* | 26.38 | < 0.0001 | **** |  |
|  | *Hsp83^j5c2^/ Hsp83^08445^* | 30.20 | < 0.0001 | **** |  |
| *Hsp83^e6A^/+* | *Hsp83^e6A^/ Hsp83^08445^* | 52.41 | < 0.0001 | **** |  |
| *Hsp83^e6D^/+* | *Hsp83^e6D^/ Hsp83^08445^* | 4.87 | 0.98 | ns |  |
| *Hsp83^j5c2^/+* | *Hsp83^j5c2^/ Hsp83^08445^* | 5.71 | 0.61 | ns |  |
| *UAS-Cas9, Hsp83sgRNA* | *Hsp83sgRNA/+* | 27.43 | < 0.0001 | **** | 4 |
|  | *Clk856 Gal4>UAS-Cas9, +* | 26.63 | < 0.0001 | **** |  |
|  | *Pdf Gal4>UAS-Cas9, +* | 61.54 | < 0.0001 | **** |  |
|  | *Clk856 Gal4>UAS-Cas9, Hsp83 sgRNA* | 12.96 | <0.01 | ** |  |
|  | *Pdf Gal4>UAS-Cas9,*  *Hsp83 sgRNA* | 3.25 | 0.43 | ns |  |
| *Hsp83sgRNA/+* | *Clk856 Gal4>UAS-Cas9,+* | 1.00 | 1.0 | ns |  |
|  | *Pdf Gal4>UAS-Cas9,+* | 7.64 | 0.03 | * |  |
|  | *Clk856 Gal4>UAS-Cas9, Hsp83 sgRNA* | 72.25 | < 0.0001 | **** |  |
|  | *Pdf Gal4>UAS-Cas9,*  *Hsp83 sgRNA* | 56.60 | < 0.0001 | **** |  |
| *Clk856-Gal4> UAS-Cas9,+* | *Clk856 Gal4>UAS-Cas9, Hsp83 sgRNA* | 60.85 | < 0.0001 | **** |  |
| *Pdf Gal4 > UAS Cas9,+* | *Pdf Gal4 >UAS-Cas9,*  *Hsp83 sgRNA* | 104.29 | < 0.0001 | **** |  |
| *Clk856 Gal4 >UAS-Hsp83, Hsp83^08445^/Hsp83^08445^* | *Clk856 Gal4 > Hsp83^08445^/+* | 4.18 | 0.12 | ns | S2A |
|  | *Clk856 Gal4 > Hsp83^08445^/Hsp83^08445^* | 18.95 | < 0.0001 | **** |  |
| *Clk856 Gal4 > Hsp83^08445^/Hsp83^08445^* | *Clk856 Gal4 > Hsp83^08445^/+* | 5.61 | 0.05 | . |  |
| *Pdf^01^/+* | *Pdf^01^/ Hsp83^08445^* | 11.26 | <0.01 | ** | S2B |
|  | *Pdf^01^/ Hsp83^e6A^* | 13.17 | <0.01 | ** |  |
| *Hsp83^08445^/+* | *Pdf^01^/ Hsp83^08445^* | 49.56 | < 0.0001 | **** |  |
|  | *Pdf^01^/ Hsp83^e6A^* | 51.74 | < 0.0001 | **** |  |
| *Hsp83^e6A^/+* | *Pdf^01^/ Hsp83^e6A^* | 44.93 | < 0.0001 | **** |  |

*Note:* **** *p* < .0001, *** *p* < .001, ** *p* < .01, * *p* < .05, ns *p* >.05. p-value was adjusted using the Bonferroni method.
