# Supplementary material for: Hsp90 buffers behavioral variability by regulating Pdf transcription in clock neurons of Drosophila melanogaster
Source: PLoS Genet. 2026 Feb 17;22(2):e1012044. doi: 10.1371/journal.pgen.1012044 (PMC12952617; doi:10.1371/journal.pgen.1012044)
Supplement: S7 Table — (DOCX) [file pgen.1012044.s010.docx]

**S7 Table.** **Estimation statistics signal intensity.**

| Group | Genotype | ZT | Intensity (mean±SEM) | Difference intensity | 95%CI | | Fig. |
| --- | --- | --- | --- | --- | --- | --- | --- |
|  |  |  |  |  | low | high |  |
| s-LNv | *Pdf-Red;Hsp83^08445^* | 02 | 1.1 ± 0.2 | -1.01 | -2.01 | -0.4 | 5C |
|  | *Pdf-Red;Hsp83^08445^/+* |  | 2.1 ± 0.3 |  |  |  |  |
| Dorsal projection | *Pdf-Red;Hsp83^08445^* | 02 | 0.1 ± 0.03 | -0.09 | -0.2 | -0.01 | 5D |
|  | *Pdf-Red;Hsp83^08445^/+* |  | 0.15 ± 0.03 |  |  |  |  |
| l-LNv | *Pdf-Red;Hsp83^08445^* | 02 | 0.4 ± 0.1 | -0.6 | -1.03 | -0.3 | 5E |
|  | *Pdf-Red;Hsp83^08445^/+* |  | 1.02 ± 0.1 |  |  |  |  |
| s-LNv | *Hsp83sgRNA+* | 02 | 4.6 ± 0.40 | 5.9 | 4.3 | 7.5 | 5F |
|  |  | 14 | 10.5 ± 0.74 |  |  |  |  |
| Dorsal projection |  | 02 | 1.3 ± 0.11 | - 0.6 | - 0.9 | - 0.3 | 5G |
|  |  | 14 | 0.7 ± 0.10 |  |  |  |  |
| l-LNv |  | 02 | 13.0 ± 1.11 | 0.48 | - 2.7 | 2.9 | 5H |
|  |  | 14 | 13.4 ± 0.87 |  |  |  |  |
| s-LNv | *Clk856 Gal4>UAS-Cas9, Hsp83 sgRNA* | 02 | 9.9 ± 0.62 | - 6.3 | - 7.8 | - 4.6 | 5I |
|  |  | 14 | 3.5 ± 0.53 |  |  |  |  |
| Dorsal projection |  | 02 | 1.2 ± 0.08 | - 1.03 | - 1.2 | - 0.8 | 5J |
|  |  | 14 | 0.2 ± 0.05 |  |  |  |  |
| l-LNv |  | 02 | 10.9 ± 0.57 | - 6.5 | - 8.1 | - 4.5 | 5K |
|  |  | 14 | 4.4 ± 0.72 |  |  |  |  |
| l-LNv | *per^01^;8.0-luc;Hsp83^08445^* | 22 | 7.1 ± 1.7 | 4.2 | 0.8 | 7.8 | 6C |
|  | *per^01^;8.0-luc* |  | 2.9 ± 0.9 |  |  |  |  |
| s-LNv | *per^01^; 8.0-luc;Hsp83^08445^* | 22 | 4.3 ± 1.1 | 2.5 | 0.5 | 4.9 | 6C |
|  | *per^01^;8.0Luc* |  | 1.7 ± 0.4 |  |  |  |  |
| LNd | *per^01^; 8.0-luc;Hsp83^08445^* | 22 | 7.5 ± 1.2 | 4.7 | 2.0 | 6.7 | 6C |
|  | *per^01^; 8.0-luc* |  | 2.8 ± 0.5 |  |  |  |  |
| 5^th^ s-LNv | *per^01^; 8.0-luc;Hsp83^08445^* | 22 | 3.7 ± 1.5 | 3.4 | 1.0 | 6.6 | 6C |
|  | *per^01^; 8.0-luc* |  | 0.2 ± 0.2 |  |  |  |  |
| s-LNv | *Pdf-Red;Hsp83^08445^* | 02 | 0.4 ± 0.1 | -0.2 | -0.5 | -0.04 | S3A |
|  | *Pdf-Red;+/+* |  | 0.6 ± 0.1 |  |  |  |  |
| Dorsal projection | *Pdf-Red;Hsp83^08445^* | 02 | 0.2 ± 0.05 | -0.08 | -0.2 | 0.05 | S3A |
|  | *Pdf-Red;+/+* |  | 0.25 ± 0.03 |  |  |  |  |
| l-LNv | *Pdf-Red;Hsp83^08445^* | 02 | 0.4 ± 0.1 | -0.4 | -0.6 | -0.2 | S3A |
|  | *Pdf-Red;+/+* |  | 0.8 ± 0.1 |  |  |  |  |
| Cell bodies | *CCAP Gal4>UAS myr mRFP1;Hsp83^08445^* | 02 | 3.3 ± 0.3 | 0.2 | -1.0 | 0.4 | S3B |
|  | *CCAP Gal4>UAS myr mRFP1* |  | 3.1 ± 0.4 |  |  |  |  |
| Projections | *CCAP Gal4>UAS myr mRFP1;Hsp83^08445^* | 02 | 2.1 ± 0.2 | -0.2 | -0.9 | 0.8 | S3B |
|  | *CCAP Gal4>UAS myr mRFP1* |  | 2.2 ± 0.3 |  |  |  |  |
| s-LNv | *Hsp83^08445^* | 20 | 8.5 ± 3.4 | 3 | -2.3 | 12.4 | S3E |
|  | *iso31* |  | 5.5 ± 1.2 |  |  |  |  |
| l-LNv | *Hsp83^08445^* | 20 | 11.9 ± 3.9 | 2.9 | -4.6 | 12.1 | S3E |
|  | *iso31* |  | 9 ± 2.4 |  |  |  |  |
