# Supplementary material for: Hsp90 buffers behavioral variability by regulating Pdf transcription in clock neurons of Drosophila melanogaster
Source: PLoS Genet. 2026 Feb 17;22(2):e1012044. doi: 10.1371/journal.pgen.1012044 (PMC12952617; doi:10.1371/journal.pgen.1012044)
Supplement: S8 Table — (DOCX) [file pgen.1012044.s011.docx]

**S8 Table. Number of PER^+^ lateral neurons per hemisphere in *per^01^;8.0-luc* brains.**

| Neurons | Genotype | # hemisphere | # neurons per hemisphere (mean±SEM) |
| --- | --- | --- | --- |
| LNd | *per^0^;8.0-luc* | 8 | 3.3 ± 0.27 |
|  | *per^0^;8.0-luc;Hsp83^08445^* | 7 | 5.1 ± 0.23 |
| l-LNv | *per^0^;8.0Luc* | 8 | 1.1 ± 0.29 |
|  | *per^0^;8.0-luc;Hsp83^08445^* | 7 | 3.0 ± 0.54 |
| s-LNv | *per^01^;8.0-luc* | 8 | 0.7 ± 0.23 |
|  | *per^0^;8.0-luc;Hsp83^08445^* | 7 | 2.6 ± 0.49 |
| 5^th^ s-LNv | *per^0^;8.0-luc;* | 8 | 0.01 ± 0.01 |
|  | *per^0^;8.0-luc;Hsp83^08445^* | 7 | 0.6 ± 0.18 |
